# Voluntary and tremorogenic inputs to motor neuron pools of agonist/antagonist muscles in essential tremor patients

**DOI:** 10.1152/jn.00407.2019

**Published:** 2019-09-11

**Authors:** Gonthicha Puttaraksa, Silvia Muceli, Juan Álvaro Gallego, Ales Holobar, Steven K. Charles, Jose L. Pons, Dario Farina

**Affiliations:** ^1^Department of Bioengineering, Imperial College London, London, United Kingdom; ^2^Division of Signal Processing and Biomedical Engineering, Department of Electrical Engineering, Chalmers University of Technology, Gothenburg, Sweden; ^3^Neural and Cognitive Engineering Group, Centre for Automation and Robotics, Spanish National Research Council, Arganda del Rey, Spain; ^4^Faculty of Electrical Engineering and Computer Science, University of Maribor, Maribor, Slovenia; ^5^Department of Mechanical Engineering and Neuroscience Center, Brigham Young University, Provo, Utah; ^6^Legs & Walking AbilityLab, Shirley Ryan AbilityLab, Chicago, Illinois; ^7^Department of Physical Medicine & Rehabilitation, Feinberg School of Medicine, Northwestern University, Chicago, Illinois; ^8^Department of Biomedical Engineering and Department of Mechanical Engineering, McCormick School of Engineering, Northwestern University, Chicago, Illinois; ^9^Neural Rehabilitation Group, Cajal Institute, Spanish National Research Council, Madrid, Spain

**Keywords:** coherence, EMG, essential tremor, motor units, neural drive, voluntary control

## Abstract

Pathological tremor is an oscillation of body parts at 3–10 Hz, determined by the output of spinal motor neurons (MNs), which receive synaptic inputs from supraspinal centers and muscle afferents. The behavior of spinal MNs during tremor is not well understood, especially in relation to the activation of the multiple muscles involved. Recent studies on patients with essential tremor have shown that antagonist MN pools receive shared input at the tremor frequency. In this study, we investigated the synaptic inputs related to tremor and voluntary movement, and their coordination across antagonist muscles. We analyzed the spike trains of motor units (MUs) identified from high-density surface electromyography from the forearm extensor and flexor muscles in 15 patients with essential tremor during postural tremor. The shared synaptic input was quantified by coherence and phase difference analysis of the spike trains. All pairs of spike trains in each muscle showed coherence peaks at the voluntary drive frequency (1–3 Hz, 0.2 ± 0.2, mean ± SD) and tremor frequency (3–10 Hz, 0.6 ± 0.3) and were synchronized with small phase differences (3.3 ± 25.2° and 3.9 ± 22.0° for the voluntary drive and tremor frequencies, respectively). The coherence between MN spike trains of antagonist muscle groups at the tremor frequency was significantly smaller than intramuscular coherence. We predominantly observed in-phase activation of MUs between agonist/antagonist muscles at the voluntary frequency band (0.6 ± 48.8°) and out-of-phase activation at the tremor frequency band (126.9 ± 75.6°). Thus MNs innervating agonist/antagonist muscles concurrently receive synaptic inputs with different phase shifts in the voluntary and tremor frequency bands.

**NEW & NOTEWORTHY** Although the mechanical characteristics of tremor have been widely studied, the activation of the affected muscles is still poorly understood. We analyzed the behavior of motor units of pairs of antagonistic wrist muscle groups in patients with essential tremor and studied their activity at voluntary movement- and tremor-related frequencies. We found that the phase relation between inputs to antagonistic muscles is different at the voluntary and tremor frequency bands.

## INTRODUCTION

Tremor is characterized by rhythmic oscillations of body parts around joints (Lyons and Pahwa 2005). Whereas physiological tremor is a low-amplitude oscillation inherently present in healthy muscle activation at an approximate frequency of 10 Hz, pathological tremor has a broader frequency range (1–25 Hz) and higher oscillatory amplitude (Hess and Pullman 2012; Zhang et al. 2009). The most common pathological tremor is essential tremor (ET), affecting 4% of the population aged over 40 yr (Benito-León and Louis 2006).

ET has frequency ranging from 3 to 10 Hz (Hess and Pullman 2012; Louis 2005) and often occurs while patients are maintaining a posture of body parts against gravity (postural tremor) or during active movements (kinetic tremor). It is suggested that tremor originates from abnormal oscillations in supraspinal centers, such as in the cerebello-thalamo-cortical pathway (Benito-León and Louis 2006), potentially involving the basal ganglia (Louis 2005; Lyons and Pahwa 2005) and motor cortex, as well (Gallego et al. 2015b; Raethjen et al. 2007). These pathological neural oscillations may be amplified or attenuated by “resonance” due to the limb properties and the reflex loops (Deuschl et al. 2001; Gallego et al. 2015b; Lakie et al. 2012). The contributions of these feedback components to tremor severity are however largely unknown.

Muscle contraction is ultimately caused by the output from the pools of spinal motor neurons (MNs). Therefore, the accurate identification of the timings of MN activity provides an important window into tremor pathophysiology (Gallego et al. 2011; Holobar et al. 2011). The discharge timings of MNs can be assessed in vivo during natural movements by decoding electromyographic (EMG) signals into motor unit (MU) firings (MU spike trains) (De Luca and Forrest 1972; Merletti and Farina 2009; Muceli et al. 2015). Recently, methods for MN analysis have been substantially advanced by techniques based on high-density surface EMG electrodes (Drost et al. 2006; Farina et al. 2016; Merletti et al. 2008) and blind decomposition of the resulting multichannel EMG recordings (Chen et al. 2016; Holobar and Zazula 2003, 2007; Negro et al. 2016). These approaches allow identification of the activity of relatively large MN populations.

In this study, we investigated the neural control of agonist/antagonist muscles in ET by assessing the discharge timings of MNs and estimating the synaptic inputs that determined their activities. The MN pool of a muscle receives inputs that are shared by all MNs (common inputs) and inputs independent for each MN (Farina et al. 2014; Gallego et al. 2011). The presence of common input to MNs determines correlation in their output discharges (Gallego et al. 2011, 2015b; Holobar et al. 2011; Kirkwood and Sears 1982; Negro and Farina 2011, 2012; Nordstrom et al. 1992). Therefore, the relative strength of common input to MN pools can be indirectly inferred with time-domain (synchronization) (Bremner et al. 1991; Dietz et al. 1976; Hua et al. 1998; Kim et al. 2001) or frequency-domain (coherence) (Christakos et al. 2009; Hellwig et al. 2003; Raethjen et al. 2000) correlation analysis. Coherence analysis has been previously applied to quantify the correlation between cortical inputs and spinal MN outputs (corticomuscular coherence) by using simultaneous recordings of electroencephalogram (EEG) and EMG (Hellwig et al. 2003; Raethjen et al. 2007) or MN population activity (Gallego et al. 2015b) to elucidate the origin of tremor. Recent studies also applied this analysis to pairs of MNs or groups of MNs of different muscles to reveal MN population behavior (Gallego et al. 2015a; Holobar et al. 2011) and to assess the contribution of peripheral feedback loops to tremor (Gallego et al. 2015b; Holobar et al. 2012; Raethjen et al. 2000; using surface EMG). Coherence analysis has also been used to assess the extent of common synaptic input to agonist muscles in healthy subjects (Negro and Farina 2012), revealing that only a small subset of MU spike trains is sufficient to sample the common synaptic input. However, because MUs of tremor-affected muscles fire in burst and are prone to be more synchronized than those of healthy subjects (Gallego et al. 2015b; Holobar et al. 2012), this hypothesis also has to be validated in individuals with pathological tremor.

Recent simulation and experimental studies on the phase difference (delays) between MN activities in antagonist muscles suggest that, during tremor, this phase difference is modulated by the intensity of the supraspinal tremor oscillatory input to each muscle and the relative strength of their voluntary drives (Gallego et al. 2015a). However, MN activities in the very low frequency band (1–3 Hz, voluntary drive) have not been thoroughly investigated, probably due to limitations in the coherence analysis applied to surface EMG signals (Negro et al. 2015). Thus it is necessary to identify individual MN activities to concurrently investigate synaptic inputs received by MNs in the voluntary control and tremor bands. This is supported by work showing that cumulative spike trains (CSTs) provide a more accurate estimation of the tremor-related activity than surface EMG (Dideriksen et al. 2011).

The main aim of this study is to concurrently investigate the synaptic inputs received by MNs of agonist/antagonist muscle groups for voluntary control and tremor. We hypothesized that the phase relation between inputs to the two muscles of the pair would be different between concurrent voluntary and tremor bands, suggesting that they can be modulated independently. Our results validate this hypothesis for the first time.

## MATERIALS AND METHODS

### 

#### Patients.

The data were collected from 15 ET patients (6 women; age 69.5 ± 9.9 yr) with a diagnosis of definite ET according to the criteria of the Tremor Investigation Group and the consensus of the Movement Disorder Society (Deuschl et al. 1998). According to the Fahn–Tolosa–Marin scale, the mean score of the tremor severity in the most affected limb was 32.1 ± 11.3 (range 15–50). Eight patients had left tremor predominance, and one had tremor that equally affected both limbs (bilateral).

All procedures were approved by the Ethical Committees at the University Hospital “12 de Octubre” (Madrid), which guaranteed compliance with the Declaration of Helsinki. Written (signed) informed consent was obtained from all enrollees.

#### Task.

The patients were seated on a comfortable armchair with their forearms fully supported and were asked to stretch their hands out against gravity with palms down to evoke postural tremor. This position is commonly used to assess severity of postural tremor (Bain et al. 1993). The EMG signals were recorded for 40 s to 4 min depending on the time the patient felt comfortable with the task. The EMG signals were acquired from both forearms using 4 grids of 64 surface electrodes, arranged in 13 rows and 5 columns (1 electrode missing in a corner) with 8-mm interelectrode distance. Electrode grids were placed over the wrist flexor and extensor muscles (centered above the flexor carpi radialis and extensor digitorum communis, respectively), with electrode columns approximately aligned with muscle fibers. Because the recording surface of the grid unavoidably extended beyond the two above-mentioned muscles, including other muscles, in the following we refer to them as antagonist groups of muscles responsible for wrist flexion/extension. A soaked wrist strap was worn and acted as a common reference. Before the electrode grids were attached, the recording area on the forearms was shaved, lightly rubbed using abrasive paste, and cleansed with water. Analog EMG signals recorded from the electrode grids were amplified as bipolar recordings along the direction of the fibers and were bandpass filtered (EMG-USB2; OT-Bioelettronica, Italy) between 10 and 750 Hz before being converted to digital signals at a 2,048-Hz sampling frequency by a 12-bit analog-to-digital converter. The data were stored in a database for further offline analysis using MATLAB.

#### EMG decomposition.

The convolution kernel compensation algorithm (Holobar et al. 2010; Holobar and Zazula 2003, 2007) was used to discriminate the spike train of each MU and to express it as a sequence of binary values (0 or 1) at the MN discharge times. The decomposition accuracy was estimated by using the pulse-to-noise ratio (PNR) (Holobar et al. 2014). Only the recordings from which at least two MUs were identified with PNR > 26 dB and firing in a time interval ≥30 s (see below) were retained for further analysis. The MU spike trains from forearms with predominant tremor (2 muscle groups) of 14 patients fulfilled these criteria and were included in the analysis. To increase the sample size, we included an additional patient who had bilateral tremor with MU spike trains from three muscle groups (flexor and extensor muscles of the left wrist and extensor muscles of the right wrist) meeting the criteria. MU spike trains were therefore extracted from 31 muscle groups. The time interval in which the firing rate of each MU was ≥60% of the maximum firing of the same MU was selected for further analysis. This corresponded to 30 s for 11 patients and 60 s for the remaining 4 patients.

#### Pools of motor units.

The neural drive to a muscle was represented by the pooled cumulative discharges of all the MUs whose spiking activity was detected (Farina et al. 2014; Gallego et al. 2015a). This summation of the spike trains is called CST. For the intramuscular correlation analysis, the MUs detected in a muscle were divided into two groups with the sizes of maximum half number of MUs (i.e., if 11 MUs were detected, they were divided into 2 CSTs comprising 5 MUs and 6 MUs) that were summed to create two CSTs. Similarly, for the intermuscular correlation analysis, the two CSTs were generated from all the activated MUs in the flexor and extensor muscles, respectively. For this analysis, the maximum number of MUs in each CST depended on the smaller number of MUs of either extensor or flexor muscles. Every possible combination of MU grouping to generate the CSTs was analyzed, and the results were averaged.

#### Tremor and voluntary drive.

Coherence analysis was performed on the CSTs using nonoverlapping Hanning windows with 0.125-Hz resolution. Coherence was calculated according to the following equation:

(1) $$Cxy(f)=|Gxy(f){|}^{2}/\left[Gxx(f)Gyy(f)\right],$$

where *Gxy*(*f*) is the cross-spectral density between the CSTs *x* and *y*, *Gxx*(*f*) and *Gyy*(*f*) are their corresponding autospectral densities, and *f* is the frequency of interest. The peak frequency in the coherence spectrum within the tremor (3–10 Hz) and voluntary drive (1–3 Hz) frequency ranges were considered as the dominant tremor frequency and dominant voluntary drive frequency, respectively. The dominant tremor frequency represents the frequency of the tremor oscillator that is common to the MN pool (Gallego et al. 2015a).

#### Phase difference between neural drives.

To calculate phase differences at the tremor frequency, we filtered the CSTs with a 2-Hz-wide bandpass filter centered at the dominant tremor frequency (3rd-order zero-lag Butterworth filter) before computing instantaneous phases of each CST using the Hilbert transform. The average difference between the instantaneous phase of pairs of CSTs represents the phase difference between their neural drive. The same procedure was performed at the frequency of 1–3 Hz to reflect the MU activities at the voluntary drive frequency. This small bandwidth was chosen because it enhances sampling efficiency (Negro and Farina 2012). Circular phase histograms of the instantaneous phases were produced with resolution of 20° per bin, and the mean delays between neural drives were calculated by the following equation:

(2) $$delay=\frac{\bar{\left|\phi_{\text{nd}}\right|}}{2\pi f_{i}},$$

where $\bar{\left|\phi_{\text{nd}}\right|}$ is the absolute circular mean of the instantaneous phase difference, and *f_i_* = *f*_tr_ and *f_i_* = *f*_vol_ are the tremor and voluntary drive frequency, respectively.

#### Shared common synaptic input.

The coherence analysis was performed with MU spike trains separately at the voluntary drive and tremor frequencies to investigate whether there were shared intra- and intermuscular synaptic inputs in those relevant frequency ranges. The coherence analysis was applied to all pairs of CSTs, generated as described above. For each patient, the autospectral density and cross-spectral density were computed for determining the coherence value. The confidence level (CL) of each coherence profile was calculated by the following equation (Rosenberg et al. 1989):

(3) $$CL=1-(1=\alpha )\frac{1}{N-1},$$

where *N* is the number of data segments and α is the level of confidence, which was set to 0.95. This implies that the coherence values were considered significant if they were greater than 95% of CL.

#### Proportions of common synaptic input.

The coherence function provides an indication of the extent of tremor input that is sampled by the MUs. To study the strength of common input to the MNs, we investigated changes in the coherence magnitude at the tremor and voluntary drive frequencies as a function of the number of MUs in CSTs. For this purpose, we created CSTs by randomly selecting *k* of all the MUs identified for each muscle, with *k* varying from one to the maximum number of MU spike trains detected in that muscle.

#### Statistical analysis.

Normality of the data was visually observed and assessed using the quantile-quantile plot and the Shapiro–Wilk test, respectively. Pairs of normally distributed variables were compared using paired *t* tests, whereas the Mann–Whitney–Wilcoxon test was used to compare nonnormal data. The correlations between two variables were measured using Pearson’s correlation test for normally distributed data and Spearman’s correlation test for nonnormal data. We investigated how the coherence peak value (in the voluntary drive and tremor frequency bandwidths) changed as a function of the number of MUs used in the analysis using either ANOVA or the Kruskal–Wallis test, depending on the data distribution, followed by pairwise comparisons to compare individual differences. Four separate analyses were performed for intramuscular and intermuscular coherence in the voluntary and tremor frequency bands. Also, for the voluntary and tremor bands separately, we compared the intramuscular and intermuscular coherence with the same number of MUs. In all cases, the null hypothesis was rejected for *P* values <0.05. The results are means ± SD.

## RESULTS

### 

#### Motor unit activities.

**Fig. 1.**
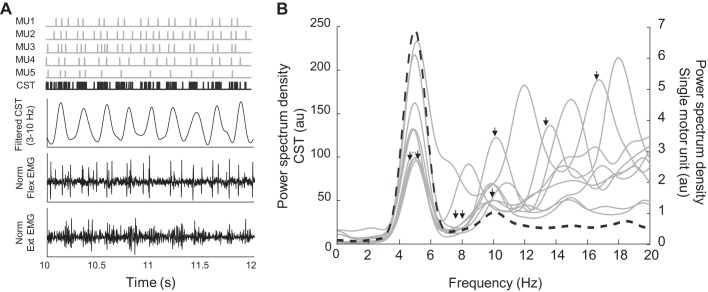
Comparison of the power spectrum density (PSD) of single-motor unit (MU) spike trains and cumulative spike train (CST). *A*: representative example of 5 MU spike trains (gray) and the CST (black), the CST bandpass filtered at the tremor frequency (3–10 Hz), and the central electromyographic (EMG) channel of the surface grid positioned over the flexor (Flex) and extensor (Ext) muscle groups, respectively. *B*: the PSDs of each spike train (gray solid lines) and the PSD of their CST (black dashed line). The PSDs of the individual MUs showed high power at the frequency of discharge (black arrows), the tremor frequency (3–10 Hz), and its harmonics, whereas the PSD of the CST showed a single peak at the tremor frequency reflecting population coding of the MUs. au, Arbitrary units; Norm, normalized.

The total number of MU spike trains that were analyzed from the 15 subjects was 211 (6.8 ± 4.0 MUs per muscle group). Average discharge rate was 14.3 ± 5.0 Hz. Power spectral densities (PSDs) of the MU spike trains were computed to identify the tremor frequencies. Figure 1*A* illustrates a subset of the identified MU spike trains and their CST for one muscle group from a representative subject. The PSDs of each MU and of the CST are compared in Fig. 1*B*. The PSDs of the individual MUs showed high power at the frequency of discharge, the tremor frequency (5.6 ± 1.8 Hz) and its harmonics. Conversely, the PSD of the CST showed a single peak at the tremor frequency (5.0 Hz). Across patients, the average frequency of the power peak for the CST was 6.9 ± 1.8 Hz, ranging from 4.8 to 10.0 Hz (2 muscle groups × 11 patients and 3 muscle groups × 1 patient). In most patients (11 of 15), the MUs in at least one group of muscles also showed a power peak at the voluntary drive frequency (not evident in the example represented in Fig. 1*B*), and in 5 of 31 cases, the peak of the power spectrum at the voluntary drive frequency was higher than the peak at the tremor frequency.

**Fig. 2.**
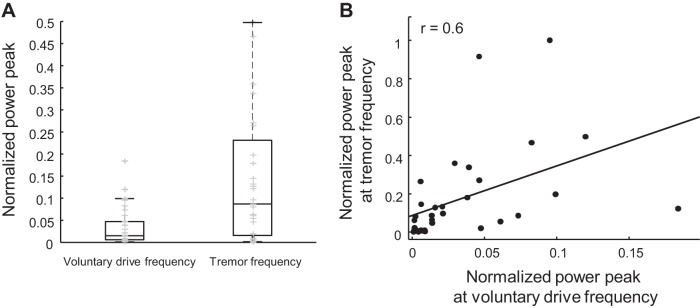
Comparison between power peak at the voluntary drive and tremor frequencies and their correlation. The power peaks were normalized with respect to the maximum peak across patients. *A*: the boxplot shows that the normalized power peaks at the voluntary drive frequency were lower than those at the tremor frequency. Two outliers at 0.9 and 1 on the boxplot of the tremor frequency are not visible because the *y*-axis was limited to 0.5 for better visualization. Gray symbols represent the power peak of each patient. *B*: the scatter plot shows that these peaks from two frequency bands were correlated, indicating the influence of the strength of the voluntary command on the strength of the tremor.

The normalized power peaks of the CST at the voluntary drive and tremor frequencies were also compared to investigate whether the voluntary drive could potentially contribute to the tremor input. The Mann–Whitney–Wilcoxon and Spearman’s correlation tests were used to search for associations between the power peaks. The results showed that the power peak at the voluntary drive was statistically lower than the power peak at the tremor frequency during postural tremor (*P* < 0.001; Fig. 2*A*). Moreover, the peaks in the two frequency bands showed significant positive correlation (*r* = 0.6, *P* < 0.001, Spearman’s rank correlation; Fig. 2*B*).

#### Common synaptic inputs to motoneuron pools.

**Fig. 3.**
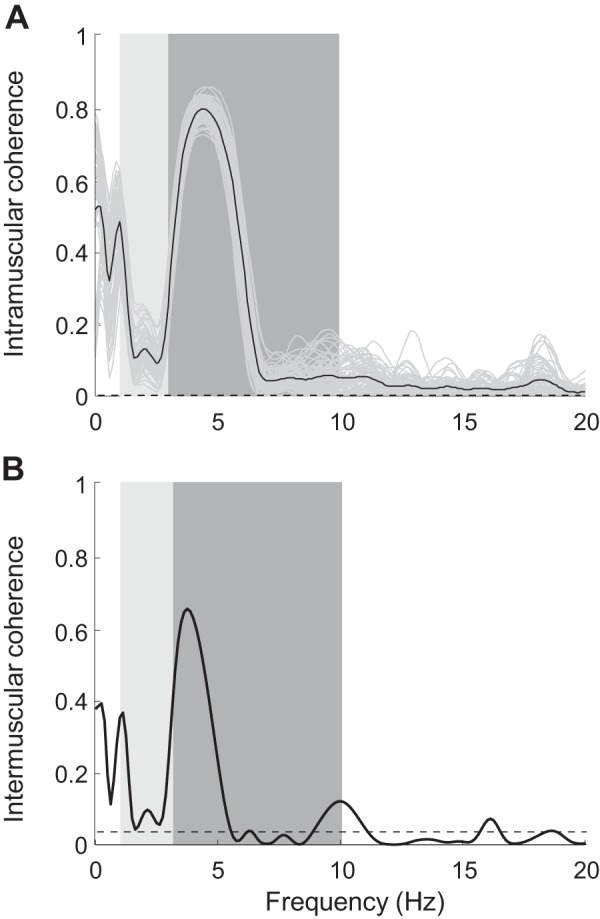
Representative example of intramuscular and intermuscular coherence at the tremor frequency of a patient with essential tremor (ET). *A*: intramuscular coherence of extensor muscles. Coherence between combinations of cumulative spike trains (CSTs) containing half the maximum number of motor units (Mus) detected within the muscle (gray lines) and the averaged intramuscular coherence (black line) is shown. The total number of MUs detected from extensor muscles was 7; therefore, pairs of CSTs of 3 MUs and 4 MUs were used for coherence analysis in this representative example. *B*: intermuscular coherence of the antagonist groups of muscles in the left wrist. Black dashed line is the confidence limit. Light gray and dark gray bands indicate the voluntary drive and tremor frequencies, respectively. Both intramuscular and intermuscular coherence graphs contain significant peaks at the tremor frequency and smaller peaks at the voluntary drive frequency. However, these peaks in the intermuscular case (*B*) are smaller, indicating the reduction in correlation of MU spike trains of antagonist groups of muscles.

We first investigated whether all recorded muscles received a common tremor-related synaptic input (Gallego et al. 2015b). Table 1 summarizes the results on common synaptic input to a muscle and shared common input across muscles. Figure 3*A* illustrates a representative example of common synaptic input to a muscle at the voluntary drive and tremor frequencies. From the group data analysis, the coherence peaks of all pairs of spike trains in each muscle averaged 0.2 ± 0.2 at the voluntary drive frequency and 0.6 ± 0.3 at the tremor frequency (Table 1). The average intramuscular coherence, calculated from all permutations of the CSTs within agonist muscles, showed relatively high peak within the tremor frequency band (see representative example in Fig. 3*A*). Therefore, all examined muscles received a tremor-related common synaptic input. The intramuscular coherence was significantly lower at the voluntary drive frequency compared with the tremor frequency band (Fig. 3*A*; *P* < 0.001, Mann–Whitney–Wilcoxon test).

**Table 1.** Summary of analysis results

	Intramuscular		Intermuscular	
	Voluntary (1–3 Hz)	Tremor (3–10 Hz)	Voluntary (1–3 Hz)	Tremor (3–10 Hz)
Magnitude of coherence peak				
Mean ± SD	0.24 ± 0.16	0.63 ± 0.30	0.12 ± 0.08	0.44 ± 0.28
Range	0.04–0.52	0.04–0.95	0.03–0.27	0.06–0.88
Frequency of coherence peak, Hz				
Mean ± SD	0.46 ± 0.62	6.34 ± 1.43	0.05 ± 0.03	5.75 ± 1.58
Range	0.00–3.00	3.65–10.00	0.00–0.12	3.13–8.13
Phase difference, °				
Mean ± SD	3.34 ± 25.25	3.89 ± 22.05	0.59 ± 48.79	126.9 ± 75.60
Range	−96.54 to 92.94	−92.79 to 92.01	−153.51 to 93.28	−174.73 to 177.33
Delay, ms				
Mean ± SD	4.31 ± 8.71	4.86 ± 9.94	18.08 ± 17.84	50.33 ± 34.83
Range	0.02–34.99	0.04–48.68	0.59–65.60	3.08–120.45

Data are means ± SD and ranges of coherence values, frequencies at coherence peak, phase difference between neural drive to muscles, and delays. The analysis was performed between motor units (MUs) within a muscle (intramuscular) and between MUs within antagonist muscle groups (intermuscular) at voluntary drive (1–3 Hz) and tremor (3–10 Hz) frequencies.

Similarly, we found that all groups of antagonistic muscles received a shared common synaptic input at the tremor frequency, as indicated by the coherence analysis of the CSTs from antagonist groups of muscles (intermuscular coherence, Fig. 3*B* and Table 1). The intermuscular coherence values at the tremor frequency (0.4 ± 0.3) were significantly higher than those at the voluntary drive frequency (0.1 ± 0.1; *P* < 0.001, Mann–Whitney–Wilcoxon test).

#### Delay between neural drives to muscles.

**Fig. 4.**
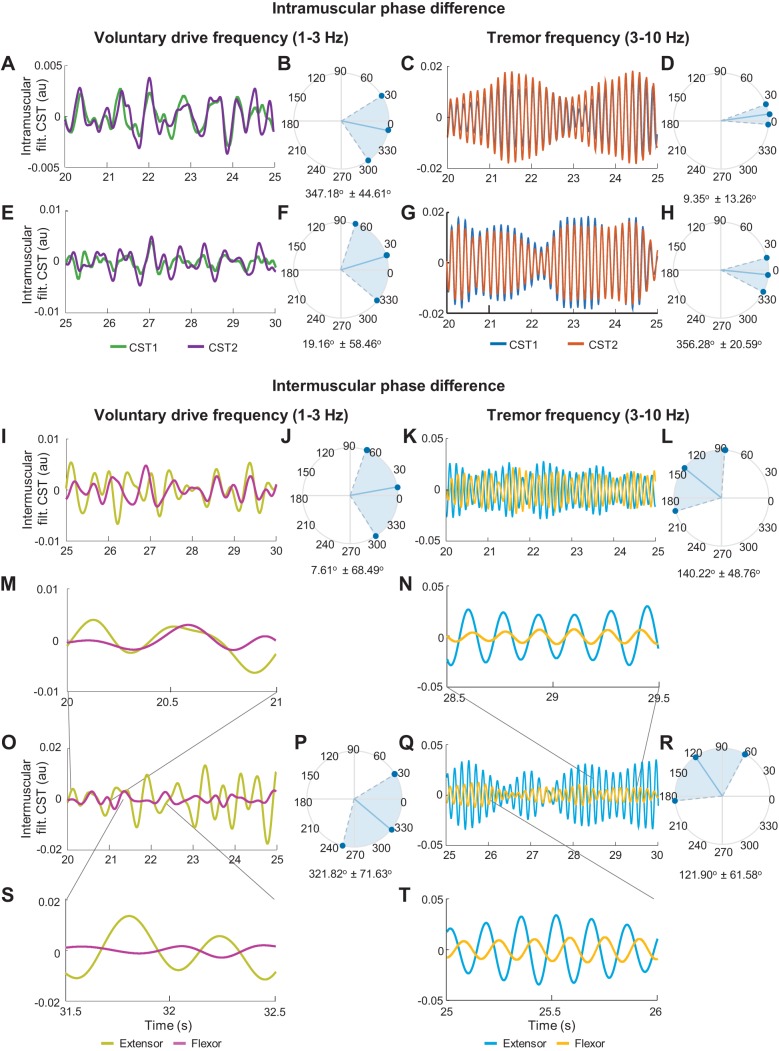
Representative phase difference between the neural drive to agonist muscles (*A–H*) and antagonist muscles (*I–T*) at the voluntary drive and tremor frequencies of a patient. *A–H*: phase difference between neural drive to extensor (*A*, *C*) and flexor (*E*, *G*) muscles of the left wrist. The phase differences were compared using the filtered cumulative spike trains [filt. CST; groups of half the maximum number of motor units (MUs)] at the voluntary drive frequency (1–3 Hz) and the tremor frequency (±1 Hz with respect to the dominant tremor frequency). *B*, *D*, *F*, and *H*: circular histograms of the intramuscular phase difference showing relatively in-phase patterns of the MU activations in both frequency ranges. *I–T*: phase difference between the neural drive to antagonist muscles of the left (*I*, *K*) and right (*O*, *Q*) wrists and their circular histograms (*J*, *L*, *P*, *R*). The average (over time) intermuscular phase difference at the voluntary drive frequency was relatively in phase (*I*, *O*), whereas that at the tremor frequency was relatively out of phase (*K*, *Q*). As shown by this representative patient data, the phase difference varied over time from in-phase to out-of-phase patterns in both frequencies (*M*, *N*, *S*, *T*). au, Arbitrary units.

**Fig. 5.**
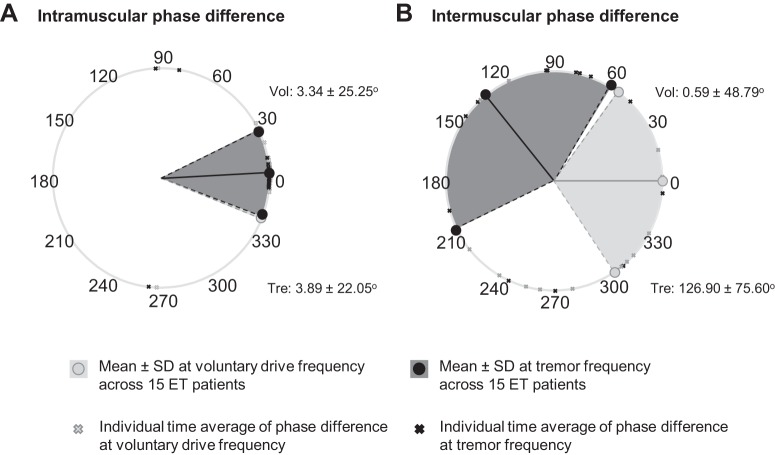
Means and variations of the phase differences between agonist (*A*) and antagonist (*B*) motor unit (MU) activations at the voluntary drive (light gray circles and light gray area) and the tremor frequencies (dark gray circles and dark gray area) of all the patients with essential tremor (ET). The cross symbols show averages over time of the phase difference of each muscle from 15 patients. During postural tremor, MUs within agonist muscles (*A*) showed small average phase difference (close to 0) with relatively narrow variation, indicating that these MUs were activated almost simultaneously at both frequencies. However, the activations of the MUs within antagonist muscles were significantly different between two frequency bands (*B*). At the voluntary drive frequency, the MUs in antagonist muscle groups showed an in-phase activation with slightly wider variation, whereas at the tremor frequency, the average phase difference increased to a higher degree (close to out-of-phase) with great variation. Vol, voluntary drive frequency; Tre, tremor-related frequency.

We investigated the temporal differences between MU activations in agonist/antagonist groups of muscles at the voluntary drive and tremor frequency bands by calculating their phase difference. The phase difference between the voluntary and tremor-related drives to pools of MUs in agonist muscles (detected by the same surface EMG grid of electrodes) showed in most cases (voluntary drive: 28 of 31 muscles; tremor: 27 of 31 muscles) an in-phase pattern (average phase difference < 10°) with relatively narrow variation. Figure 4, *A–H*, shows representative examples of the phase differences in agonist muscles for a representative patient at the voluntary drive and tremor frequencies. These were in agreement with the overall trend shown in Fig. 5*A*.

Similarly, the phase difference between voluntary drives to groups of antagonist muscles (intermuscular phase difference) were, on average, also approximately in phase (Fig. 5*B*). In stark contrast, the intermuscular phase difference at the tremor frequency showed nearly out-of-phase patterns across all sample data (Fig. 5*B*). However, the intermuscular phase difference in both frequencies often varied over time (Fig. 4, *I–T*), from almost in phase (Fig. 4, *M* and *N*) to out of phase (Fig. 4, *S* and *T*), resulting in a large spread of the circular histograms (Fig. 4, *J*, *L*, *P*, *R*). The standard deviation of the intermuscular phase difference at both frequencies was negatively correlated to the coherence values (voluntary: *r* = −0.7, *P* < 0.001, tremor: *r* = −0.8, *P* < 0.001, Spearman’s correlation test), because the larger variability in phase corresponded to lower coherence. These results show that the MNs concurrently received synaptic inputs with different phase relations between agonist and antagonist muscles.

Because the in-phase voluntary drives to agonist-antagonist muscle groups might serve to stabilize the limb, we investigated a potential association between the voluntary intermuscular coherence and tremor severity (estimated by the Fahn–Tolosa scale). However, these two variables were not correlated (*P* = 0.21 and *r* = −0.36, Spearman’s correlation test).

#### Proportion of common input.

To study the strength of common input to muscles, we investigated how coherence magnitude changed as a function of the number of MUs. We performed this analysis both within a muscle and across groups of antagonist muscles. The coherence values of all the permutations of subgroups comprising one to the maximum number of MU spike trains were computed and averaged per group.

**Fig. 6.**
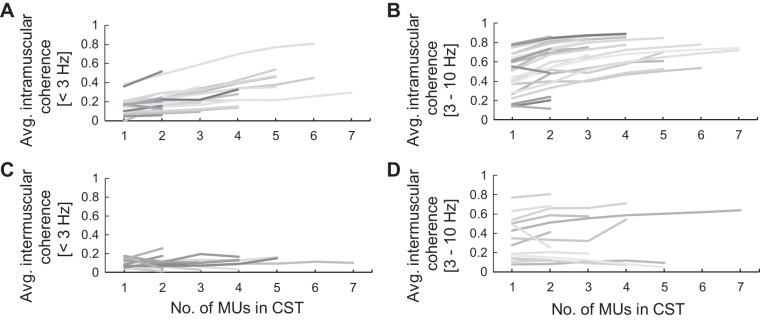
Change in magnitude of maximum coherence as a function of the number of motor units (MUs) of all patients with essential tremor (ET). *A* and *C*: intramuscular and intermuscular coherence, respectively, at the voluntary movement frequency. *B* and *D*: intramuscular and intermuscular coherence, respectively, at the tremor frequency. The intramuscular coherence values slightly increased toward plateau when more MUs were added to the cumulative spike trains (CSTs) in both frequency ranges. However, the intermuscular coherence (*C* and *D*), on average, was lower than the intramuscular coherence (*A* and *B*) in both the voluntary drive and tremor frequencies, and their increments were not statistically significant. Avg., average.

Figure 6 shows the trend of the increment in the coherence peak values due to the inclusion of additional MUs at the voluntary drive and tremor frequencies of all patients. The Kruskal–Wallis test was used to investigate the increment of the coherence values at the voluntary drive frequency because they were nonparametric, whereas ANOVA was used for the coherence values at the tremor frequency because they were normally distributed. Both statistical analyses were followed by pairwise comparisons (*t* test for normally distributed data and Wilcoxon test for nonnormal data). The intramuscular coherence values of the CSTs comprising one to four MUs at the voluntary drive and tremor frequency ranges were significantly different (*P* = 0.04 and *P* < 0.001, respectively). Pairwise comparisons revealed that the coherence of the CSTs comprising three and four MUs were significantly different from those with only one MU (voluntary drive: *P* = 0.003 and 0.001; tremor: *P* = 0.004 and 0.007, respectively), but the increment of intramuscular coherence was not significant when three-MU CSTs and four-MU CSTs were compared (*P* = 0.51 and 1.00 for the voluntary drive and tremor frequencies, respectively). Therefore, only three to four MUs were sufficient to accurately sample the voluntary and tremor inputs to agonist muscle groups. In contrast, the increase of the intermuscular coherence at both frequencies were never significant (*P* = 0.21 and 0.69, Kruskal–Wallis test and ANOVA for the voluntary drive and tremor frequency, respectively).

We then compared the intramuscular and intermuscular peak coherence values of the CSTs comprising one to four MUs. We found that, for the voluntary drive frequency, the intermuscular coherence values were lower than the intramuscular coherence values when more than two MUs were considered (*P* = 0.007 and 0.007 for 3 and 4 MUs, respectively; Kruskal–Wallis test). For the tremor frequency, the intermuscular coherence values were lower than those of the intramuscular case even when only two MUs were included in the analysis (*P* = 0.004, *P* < 0.001, *P* = 0.001 for 2, 3, and 4 MUs, respectively; ANOVA).

The relative increment in coherence values for each MU added in the calculation of the CST were also calculated to estimate their trends. The averaged relative increment of the intramuscular coherence at voluntary drive and tremor frequencies monotonically decreased from 27.7% to 3.3% and from 33.6% to 11.2% when the number of MUs increased from one to seven, respectively (Fig. 6, *A* and *B*). For the coherence values between groups of antagonist muscles (Fig. 6, *C* and *D*), the relative increment of the intermuscular coherence values did not decrease monotonically. Together, these results indicate that common inputs are less shared across antagonist muscles than they are across agonist muscles and that there may be differences between the voluntary and tremor drives in the extent of their sharing.

## DISCUSSION

In this study, we investigated the behavior of MUs in agonist/antagonist groups of muscles of 15 patients with ET during postural tremor, focusing on the relation between their voluntary and tremor-related inputs. We found that, within each muscle, coherence peaks at the voluntary drive frequency were lower than at the tremor frequency (Fig. 3*A*), likely reflecting that voluntary inputs are less shared across motor neurons than tremor-related inputs. Coherence values between groups of MUs from antagonist muscles were smaller, suggesting that motor neurons innervating antagonist muscle groups may be influenced by common inputs and also by independent inputs projected individually to each MN pool. We also observed that through the task, the relative phase of MUs from antagonist muscles varied randomly from in phase to out of phase at both the voluntary drive and tremor frequencies. However, the phase difference at the voluntary drive frequency was, on average, lower than at the tremor frequency, perhaps reflecting muscle co-contraction to maintain postural position. Taken together, these results imply that in patients with ET, MUs in antagonist muscle groups were activated differently in different frequency bands.

### 

#### Tremor characteristics.

During tremor, the MNs fire in discrete patterns with an occurrence of short interspike intervals (paired or tripled discharges; Fig. 1*A*) (Dietz et al. 1976; Holobar et al. 2012), unlike MU spike trains of healthy individuals that fire more regularly. Although the power spectrum of a single MU showed several peaks at various frequencies (Fig. 1*B*), all of them exhibited a peak in the tremor frequency band (3–10 Hz) and, in some patients also in the voluntary drive frequency band (1–3 Hz). When these MUs were pooled together to create CSTs, the spectral peaks at the voluntary drive and tremor frequencies were enhanced, whereas the power of other frequency components was reduced. This demonstrated an averaging process of the neural outputs where only the common inputs to the pools of MNs were emphasized (population coding) (Farina et al. 2016; Negro and Farina 2012). In fact, MN populations (CST) sample the input signals at a higher rate compared with single MUs and, therefore, optimize the neural input detection. The existence of a significant peak at the tremor frequency extracted from the CST (Fig. 1*B*) indicates that the MN pools received strong neural input at this frequency (Farina et al. 2014). Apart from the peaks at the voluntary drive and tremor frequencies, the PSDs showed smaller peaks in the first harmonic of the tremor, previously attributed to mechanical resonance (Raethjen et al. 2009).

The two main peaks in the PSDs of the CSTs were at the voluntary drive and tremor frequencies, but their magnitudes varied among patients. In some cases, the peak of PSDs at the voluntary drive frequency was greater than the tremor peak. However, the greater the voluntary contraction, quantified by the power peak at the voluntary drive frequency, the higher the tremor power. This positive correlation may indicate an interdependency of the tremor and voluntary drives. In fact, Schmied and Descarreaux (2010) suggested that contributions of common inputs can be enhanced by stronger voluntary contractions: higher force would increase the number of MNs recruited, and these MNs would sample better the tremor-related neural input. However, even though there was a statistically significant association between voluntary and tremor power, the model did not capture a lot of variance, weakening this interpretation.

#### Common input drive to agonist and antagonist groups of muscles.

MNs receiving the same inputs have high statistical tendency to discharge synchronously, leading to an increase in correlation between neural output of the MNs (MU spike trains). Specifically, the shared synaptic inputs equally enhance electrical potential at the membrane of the neurons receiving them, which increases their probability of firing simultaneously (de la Rocha et al. 2007; Negro and Farina 2011; Raethjen et al. 2007). This correlation in MU activations can be quantified using coherence analysis (Farina et al. 2016; Negro and Farina 2011, 2012). In turn, the measured CST coherence values reflect the strength of the shared neural drives (Christakos et al. 2009; Gallego et al. 2015a). The relatively high intramuscular coherence and in-phase pattern of the MU activations observed at the voluntary drive and tremor frequencies (Figs. 3*A* and 5*A*) thus indicate that MNs within agonist muscles shared a large amount of both inputs (Gallego et al. 2015a).

The tremor movements were mainly controlled by common inputs, which possibly include central oscillators in the primary motor cortex, pontomedullary reticular formation, and cerebellum given that these neural commands oscillate at an approximate frequency of 3–10 Hz (Williams et al. 2010). The tremor-related input was partly modulated by the level of force exertion (voluntary command). Although significant tremor peaks were also present in the intermuscular coherence spectra (Fig. 3*B*), indicating the presence of common tremor-related inputs to MNs of antagonist muscle groups, the averaged intermuscular coherence was lower compared with the intramuscular coherence, reflecting the presence of other inputs independently projected to the MN pools of the two muscle groups (Farina et al. 2016; Lemon 2008). These inputs could relate to afferent feedbacks from the muscle spindles in the antagonist muscle, which are activated when the muscle rapidly changes its length. These oscillations create resonance (Deuschl et al. 2001; Lakie et al. 2012), which corresponded to the harmonic frequency found in the PSDs of the individual MUs.

Our phase difference analysis also suggested that voluntary and tremor-related inputs could influence muscle contraction independently from each other: MU activity at the voluntary frequency tended to have small averaged phase difference (in phase: phase difference < 10°), likely reflecting muscle co-contraction (Fig. 4, *I*, *J*, *O*, *P*, and Fig. 5). Based on the assumption that the tremor oscillations are also transmitted to the antagonist muscle via afferent feedbacks in opposite phase, the phases of the muscle activations could increase toward 180° due to out-of-phase activations. This behavior can be observed in Fig. 4, *K*, *L*, *Q*, *R*, and Fig. 5, where the phase difference of antagonist MU spike trains at the tremor frequency increased. The difference in phase values within two bandwidths shows that MNs of agonist-antagonist muscle pairs may concurrently present in-phase and out-of-phase oscillatory components during the same motor task.

In addition, we observed that the phase differences in both frequencies varied greatly during the recording time (Fig. 4, *J*, *L*, *P*, *R*). This was possibly due to the instability of limb movements determined by the stretch reflex arc and mechanical resonance of the limb (Gallego et al. 2015a; Raethjen et al. 2000; Zhang et al. 2009). This MU firing modulation leads to variations in MU discharge patterns and inconsistent phase differences over time. This variation of the phase difference over time could explain the limited effectiveness of tremor suppression strategies that apply out-of-phase stimulation to antagonist muscle groups (Dosen et al. 2015; Popović Maneski et al. 2011).

#### Saturation in coherence value as a measure of shared input.

Theoretical and experimental analyses (Negro and Farina 2011, 2012) show that when the number of activated MNs is sufficient and the sampling rate is high enough to effectively sample the common input, linear transmission of this common input will saturate (Farina et al. 2014). We have shown that the saturation in the coherence values at the tremor frequency can be observed by just examining the MU activity itself. The intramuscular coherence values monotonically increased (Fig. 6, *A* and *B*), and the increment was progressively smaller with increasing numbers of MUs. In fact, the coherence values of three-MU CSTs and four-MU CSTs were not statistically different, suggesting the saturation stage of the coherence peak. This saturated coherence value can be interpreted as the true strength of the common tremor-related inputs (Farina and Negro 2015; Negro and Farina 2011, 2012). This intramuscular coherence probably does not saturate at 1 for a number of reasons, including that we recorded from several agonist muscles (not just one), that motor neurons may not be perfectly linear, that there are likely other sources of common input, e.g., from spinal afferents (Gallego et al. 2015b), and the presence of independent inputs.

#### Limitations.

Our study patients held their hands outstretched against gravity with the forearms fully supported. Given this limited effort, it is expected that fatigue did not play a major role. However, further studies are needed to investigate how our phase difference and coherence results may change at higher force levels, since in healthy individuals, a fatiguing contraction involves differential control of agonist and antagonist muscles (Lévénez et al. 2008). Also, it would be relevant to examine whether the results change depending on the hand position (e.g., with the hands in a palms-up position).

#### Conclusion.

The study of coherence between MU spike trains of patients with ET indicated that both the voluntary drive- and tremor-related inputs are common to antagonist muscle groups during postural tremor. When patients were maintaining posture, the MNs in these muscles were activated simultaneously and predominantly in an in-phase pattern at the voluntary frequency band. However, the time shifts (phase differences) in these MN activations increased, which may be due to the projection of tremor-related input transmitted through afferent feedbacks in an out-of-phase pattern. These shifts in the MN activations could be modulated by *1*) level of force exertion that determines the sampling of tremor-related input to each muscle and *2*) influences of the afferent feedbacks (resonance).

## GRANTS

This study was supported by European Projects EXTEND Grant H2020-ICT-23-2017-779982 and NeuroTREMOR Grant ICT-2011.5.1-287739. G. Puttaraksa was supported by the Royal Thai Government Scholarship. J. A. Gallego received funding from Community of Madrid Atracción de Talento Grant 2017-T2/TI C-5263. A. Holobar was supported by Slovenian Research Agency Program Funding P2-0041 and Projects J2-7357 and L7-9421.

## DISCLAIMERS

The funding agencies were not involved in the collection, analyses, and interpretation of data or writing and publication of this article.

## DISCLOSURES

No conflicts of interest, financial or otherwise, are declared by the authors.

## AUTHOR CONTRIBUTIONS

G.P., S.M., J.Á.G., and D.F. conceived and designed research; J.Á.G. and A.H. performed experiments; G.P., S.M., and A.H. analyzed data; G.P., S.M., S.K.C., and D.F. interpreted results of experiments; G.P. and S.M. prepared figures; G.P., S.M., and D.F. drafted manuscript; G.P., S.M., J.Á.G., A.H., S.K.C., J.L.P., and D.F. edited and revised manuscript; G.P., S.M., J.Á.G., A.H., S.K.C., J.L.P., and D.F. approved final version of manuscript.
